# Redox-Responsive Manganese Dioxide Nanoparticles for Enhanced MR Imaging and Radiotherapy of Lung Cancer

**DOI:** 10.3389/fchem.2017.00109

**Published:** 2017-12-04

**Authors:** Mi Hyeon Cho, Eun-Seok Choi, Sehee Kim, Sung-Ho Goh, Yongdoo Choi

**Affiliations:** ^1^Biomarker Branch, National Cancer Center, Goyang, South Korea; ^2^Therapeutic Target Discovery Branch, National Cancer Center, Goyang, South Korea

**Keywords:** manganese dioxide nanoparticles, glutathione, lung cancer, gefitinib, radiotherapy, magnetic resonance imaging

## Abstract

In this study, we synthesized manganese dioxide nanoparticles (MnO_2_ NPs) stabilized with biocompatible polymers (polyvinylpyrrolidone and polyacrylic acid) and analyzed their effect on non-small cell lung cancer (NSCLC) cells with or without gefitinib resistance *in vitro*. MnO_2_ NPs showed glutathione (GSH)-responsive dissolution and subsequent enhancement in magnetic resonance (MR) imaging. Of note, treatment with MnO_2_ NPs induced significant cytotoxic effects on NSCLC cells, and additional dose-dependent therapeutic effects were obtained upon X-ray irradiation. Normal cells treated with MnO_2_ NPs were viable at the tested concentrations. In addition, increased therapeutic efficacy could be achieved when the cells were treated with MnO_2_ NPs in hypoxic conditions. Therefore, we conclude that the use of MnO_2_ NPs in MR imaging and combination radiotherapy may be an efficient strategy for the imaging and therapy of NSCLC.

## Introduction

The incidence of lung cancer has shown a consistent increase from to 2015. In 2015, despite elaborate efforts to detect cancers at early stages, the incidence of 2005 tracheal, bronchus, and lung cancers was 2 million (95% UI) and was the leading causes of cancer-related deaths worldwide, accounting for 1.7 million deaths (Fitzmaurice et al., 2017). Non-small cell lung cancer (NSCLC) is the most common subtype of lung cancer, and accounts for more than 85% of all cases (Wangari-Talbot and Hopper-Borge, 2013). Studies have shown that 40–80% of NSCLC patients overexpress epidermal growth factor receptor (EGFR), which plays an important role in growth, survival, and chemotherapy resistance (Herbst and Shin, 2002). Recent clinical trials have shown that EGFR-tyrosine kinase inhibitors (EGFR-TKIs) such as gefitinib and erlotinib represent the best first-line treatment options for patients with EGFR mutations. EGFR-TKI therapy resulted in significant improvement in the response rate and progression-free survival when compared to conventional platinum-based combination chemotherapy (Mok et al., 2009; Maemondo et al., 2010; Mitsudomi et al., 2010). However, most patients with EGFR mutations who were treated with EGFR-TKIs acquired resistance within 9–14 months (Morgillo et al., 2016). Therefore, the development of effective therapeutic agents and methodologies to selectively treat NSCLC is an urgent, unmet clinical need.

Radiation therapy is used as the first-line treatment in more than 50% of patients with local, advanced solid tumors. It is an efficient, alternative way to avoid chemotherapeutic resistance and to eradicate tumor masses. Ionizing radiation produces highly reactive oxygen species (ROS, such as •OH) via radiolysis of H_2_O molecules in the cells, and these ROS can cause various lesions in DNA. Tumors acquire resistance to radiation therapy by increasing intracellular levels of glutathione (GSH), which can neutralize intracellular ROS by donating hydrogen (Bump and Brown, 1990). Another factor that affects the efficacy of radiation therapy is the relative levels of oxygen in the tumor. Irradiation of oxygen enhances the formation of DNA double-strand breaks and the generation of ROS. However, rapid proliferation of tumor cells and an insufficient supply of oxygen inside tumor tissues results in poor oxygenation (i.e., hypoxia) in many types of cancers. Hypoxic tumor cells are known to be two to three times more resistant to radiation than normoxic cells, and they have also been shown to possess more aggressive phenotypes in tumor development by inducing the hypoxia inducible factor (HIF) protein, which is linked to cell proliferation signals (Teicher, 1995; Vaupel and Mayer, 2007; Rapisarda and Melillo, 2010).

Recently, manganese dioxide nanoparticles (MnO_2_ NPs) have attracted attention as a novel radiosensitizer because of their ability to catalyze the production of molecular oxygen (O_2_) from hydrogen peroxide (H_2_O_2_), thereby modulating the hypoxic condition of solid tumors and potentiating radiation therapy (Prasad et al., 2014; Gordijo et al., 2015; Abbasi et al., 2016; Song et al., 2016; Tian et al., 2017). The application of albumin-based MnO_2_ NPs showed clinical relevance by modulating tumor hypoxia and enhancing radiotherapy efficacy in xenograft tumor models of murine and human breast cancers (Prasad et al., 2014; Abbasi et al., 2016; Tian et al., 2017). In addition, Song et al. showed that hyaluronic acid-coated, mannan conjugated MnO_2_ NPs not only increased tumor oxygen levels in murine breast cancer, but also turned pro-tumorigenic M2 macrophages in the surrounding tumor microenvironment into pro-inflammatory M1 macrophages and enhanced chemotherapeutic responses (Song et al., 2016). These studies showed the beneficial effect of MnO_2_ NPs on oxygen production in *in vivo* animal studies using breast cancer models.

**Figure 1:**
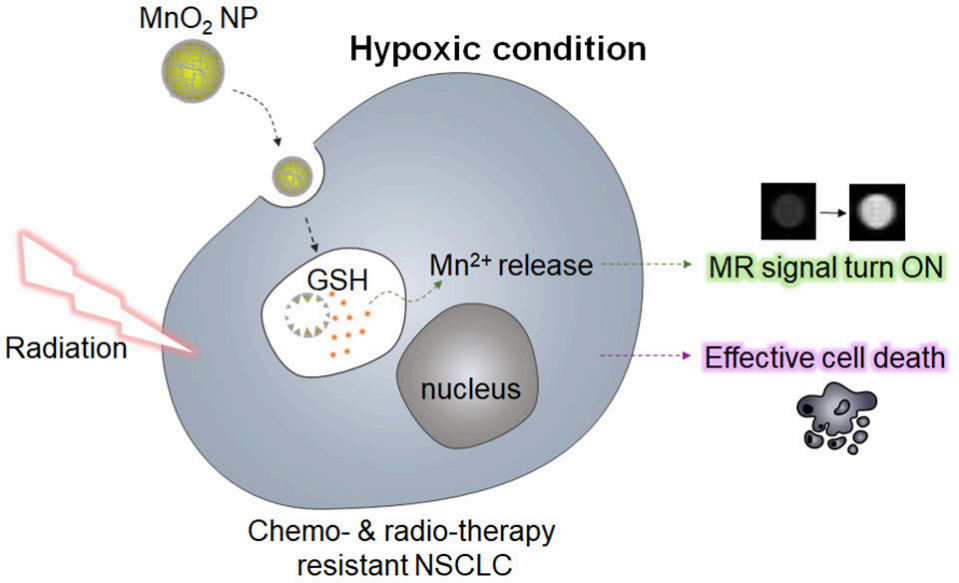
Schematic illustration of GSH-responsive MR imaging and enhanced radiotherapy.

In the present study, we synthesized MnO_2_ NPs, which were stabilized with biocompatible polymers (polyvinylpyrrolidone and polyacrylic acid), and analyzed their effect on NSCLC cells with or without gefitinib resistance (GR) *in vitro*. It has been reported that manganese dioxide (MnO_2_) can react with GSH and oxidize GSH into glutathione disulfide (GSSG) (Bump and Brown, 1990; Herszage and Afonso, 2003). Based on this property, researchers have developed MnO_2_ nanosheets or its composites for sensing GSH (Deng et al., 2011; Yan et al., 2016), and for GSH-responsive delivery of anticancer drugs (Hao et al., 2016). Therefore, we hypothesized that the use of MnO_2_ NPs may not only be useful for GSH-sensitive magnetic resonance (MR) imaging, but also for lowering the intracellular GSH levels, which would enhance the radiotherapeutic efficacy in NSCLC cells (Figure 1). Of note, we observed that treatment with MnO_2_ NPs alone induced significant cytotoxic effects on the NSCLC cells with and without GR, whereas additional therapeutic effects could be obtained upon X-ray irradiation in a dose-dependent manner. Normal cells treated with MnO_2_ NPs were viable at the tested concentrations. In addition, marked therapeutic efficacy could be observed when the NSCLC cells in hypoxic conditions were treated with MnO_2_ NPs. Therefore, we suggest that the use of MnO_2_ NPs in combination with radiotherapy may be an effective strategy for the treatment of EGFR-TKI-resistant lung cancers in normoxic and hypoxic conditions.

## Materials and methods

### Materials

Polyacrylic acid (PAA, MW 1,800), polyvinylpyrrolidone (PVP, MW 29,000), potassium permanganate (KMnO_4_), L-glutathione reduced (GSH), manganese chloride tetrahydrate (MnCl_2_·4H_2_O), 2',7'-dichlorofluorescein diacetate (DCF-DA), and cobalt (II) chloride hexahydrate (CoCl_2_·6H_2_O) were purchased from Sigma-Aldrich (St. Louis, Mo, USA). A thiol detection assay kit was purchased from Abnova (Taipei City, Taiwan). Biotech cellulose ester dialysis membrane (Spectra/Por®, molecular weight cut off 100 kDa) was obtained from Spectrum Labs (Laguna Hills, CA, USA). A CCK-8 cell viability assay kit was obtained from Dojindo (Kumamoto, Japan). A GSH-Glo kit was purchased from Promega (Madison, WI, USA). Cyclin B1 and cyclin D1 antibodies were purchased from Cell signaling Technology (Danvers, MA, USA). GAPDH antibody was purchased from Santa Cruz Biotechnology (Dallas, TX, USA). All chemicals and solvents were used without further purification.

### Methods

#### Synthesis and characterization of MnO_2_ NPs

MnO_2_ NPs were prepared by a redox reaction using KMnO_4_ and polymeric surfactant in an aqueous solution (Chen et al., 2012a,b; Koczkur et al., 2015). KMnO_4_ (31.5 mg) was dissolved in 9 mL of deionized (DI) water with magnetic stirring for 30 min at 25°C, followed by the addition of 1 mL of PVP solution (37.4 mg/mL DI water). After 15 min, 1 mL of PAA (37.4 mg/mL DI water) was rapidly added to the reaction mixture. The color of the solution changed from violet to wine in two min. Stirring was continued for an additional 15 min at 25°C. Large precipitates were removed by centrifugation at 5,000 rpm. The obtained supernatant was dialyzed [molecular weight cut-off (MWCO) 100 kDa] for 60 h to remove unreacted KMnO_4_ and free polymers, and then freeze-dried to obtain a brown powder.

The morphology of MnO_2_ NPs was observed using a transmission electron microscope (TEM, JEM 2010, JEOL Ltd., Japan). Energy dispersive X-ray spectroscopy (EDS) element mappings of MnO_2_ NPs were acquired on a JEM-F200 (JEOL Ltd., Japan). Hydrodynamic size and zeta potential of the MnO_2_ NPs were measured using Zetasizer Nano ZS-90 (Malvern Instruments, Malvern, UK). Changes in UV/Vis absorption spectra during nanoparticle synthesis were recorded by using a UV/Vis spectrophotometer (DU730, Beckman Coulter, Brea, CA, USA). The Mn content in MnO_2_ NPs was analyzed using inductively coupled plasma mass spectrometry (ICP-MS, NexION300, PerkinElmer Inc., USA). The MnO_2_ NPs, PVP, and PAA samples crushed into KBr pellets were analyzed by a Fourier-transform infrared (FT-IR) instrument (Vertex 70, Bruker), with a range of 400–4,000 cm^−1^.

#### Stability of MnO_2_ NPs at different pH conditions and in the presence of GSH

The stability of the MnO_2_ NPs at different pH conditions were evaluated by measuring changes in UV/Vis absorption spectra of the nanoparticles. MnO_2_ NPs were dispersed in either acetate buffer (pH 5.0, 0.1 M) or phosphate buffer (pH 7.4, 0.1 M) at a concentration of 80 μg/mL (200 μM Mn equivalent). UV/Vis absorption spectra of the sample solutions were recorded at 0, 5, 30, 60, and 120 min. To evaluate the reactivity of MnO_2_ NPs with GSH, MnO_2_ NPs (80 μg/mL) were dispersed in acetate buffer (pH 5.0, 0.1 M) containing 5 mM GSH. UV/Vis absorption spectra of the sample solution were recorded at 0, 5, 30, 60, and 120 min.

#### Decrease in GSH concentration by MnO_2_ NPs

We checked whether MnO_2_ NPs reduced GSH concentration in solution. Both GSH and MnO_2_ NPs were dissolved in sodium acetate buffer (0.1 M, pH 5.0) solution, and mixed together to obtain 5 mM GSH and varying concentrations of MnO_2_ NPs (0 μM, 0.25 μM, 2.5 μM, 25 μM, 250 μM, and 2.5 mM). The solutions were incubated at 25°C for 1 h to ensure completion of the reaction. The residual concentration of GSH was determined using the thiol detection kit. Fluorometric thiol detector (50 μL) was added to the diluted reaction mixture (50 μL). Fluorescence intensities of the solutions (λ_ex_ 390 nm, and λ_em_ 520 nm) were recorded on a multifunctional microplate reader (Safire 2; Tecan, Männedorf, Switzerland), and compared with a standard curve. The experiments were performed in quadruplicate.

#### *In vitro* magnetic resonance imaging (MRI)

Two hundred μL of samples of various concentrations of MnO_2_ NPs (0 mM, 0.05 mM, 0.1 mM, 0.2 mM, 0.4 mM, and 0.8 mM Mn equivalent) in phosphate buffer (pH 7.4), acetate buffer (pH 5.0), phosphate buffer containing 2 μM GSH (pH 7.4), and acetate buffer containing 5 mM GSH (pH 5.0) were prepared. MnCl_2_ aqueous solutions with different concentrations were used as the positive control. *In vitro* MR imaging experiments were performed on a 7.0-Tesla magnetic resonance imaging (MRI) system (Bruker BioSpin MRI GmbH, Germany). The imaging parameters of T1 were as follows: echo time [TE] = 9 ms, repetition time [TR] = 200 ms, and slice thickness = 2.0 mm. The imaging parameters of T2 were as follows: TE = 46 ms, TR = 3.0 s, and slice thickness = 2.0 mm. T1 and T2 values were determined for each sample as a function of concentration.

#### Cell culture

HDMVECn (human primary dermal microvasculature endothelial cells from neonatal foreskin), Raw264.7 (murine macrophage) cells, and PC9 (human NSCLC) cells were obtained from ATCC (American Type Culture Collection, Manassas, VA, USA). Gefitinib-resistant PC9 cells (PC9GR) were acquired by long-term exposure of parental PC9 cells to 1.0 μg/mL gefitinib. HDMVECn were cultured in vascular cell basal medium (ATCC, Manassas, VA, USA) supplemented with Microvascular Endothelial Cell Growth Kit-VEGF (ATCC), 10% fetal bovine serum (FBS) (Corning, Manassas, VA, USA), and penicillin–streptomycin (Invitrogen, Carlsland, CA, USA). Raw264.7 was cultured in Dulbecco's modified Eagle's medium (DMEM) supplemented with 10% FBS and penicillin–streptomycin. PC9 and PC9GR cells were maintained in RPMI1640 media (Corning) supplemented with 10% FBS and penicillin–streptomycin in a 5% CO_2_ incubator at 37°C.

#### Cytotoxicity of MnO_2_ NPs against normal and NSCLC cells

Cytotoxicity of MnO_2_ NPs was tested in normal cells (HDMVECn and Raw264.7) and NSCLC cells (PC9 and PC9GR). Briefly, cells were seeded in 96-well plates at a density of 5,000 cells/well and incubated overnight for cell attachment. The cell culture medium was replaced with fresh media containing various concentrations of MnO_2_ NPs (0, 10, 25, and 50 μg/mL), and incubated for 3 h. After washing three times, fresh cell culture medium was added to the cells, which were incubated for an additional 72 h. Cell viability was measured using a CCK-8 assay kit. Absorbance was measured at 450 nm (reference = 650 nm) using a microplate reader (Versa max, Molecular Devices, Sunnyvale, CA, USA). Untreated cells served as 100% viability control, and the medium served as the background. Data are expressed as the mean (± SD) of three data samples.

#### Changes in intracellular GSH levels after MnO_2_ NP treatment

PC9 and PC9GR cells were seeded in 96-well plates at a density of 5,000 cells/well and incubated overnight for cell attachment. The cells were treated with fresh cell culture media containing MnO_2_ NPs for 3 h. For the hypoxia treatment group, cells were pre-treated with CoCl_2_ (100 μM) for 24 h to induce hypoxia in mammalian cell cultures (Wu and Yotnda, 2011; Lee et al., 2012), and then further incubated for 3 h in the presence or absence of MnO_2_ NPs. Cells were then washed twice with phosphate-buffered saline (PBS). For the measurement of intracellular GSH levels, luminescence from the cells was measured using the GSH-Glo Glutathione Assay kit by Infinite 200 Pro (TECAN, Männedorf, Switzerland). All the reactions were carried out in triplicate.

#### *In vitro* therapeutic efficacy after X-ray irradiation

PC9 and PC9GR cells were seeded in 96-well plates at a density of 5,000 cells/well and incubated overnight for cell attachment. The existing cell culture medium was replaced with fresh media containing various concentrations of MnO_2_ NPs (0, 10, 25, and 50 μg/mL), and incubated for 3 h. After washing three times, fresh cell culture media was added, and the cells were irradiated with X-rays (0 Gy, 1 Gy, 5 Gy, and 10 Gy) using an X-RAD 320 irradiator (Precision X-ray, North Branford, CT, USA). The cells were then incubated for 72 h, and cell viability was measured using the CCK-8 assay kit. Absorbance was measured at 450 nm (reference = 650 nm) using a microplate reader. Untreated control cells (i.e., without MnO_2_ NP treatment and X-ray irradiation) served as the 100% viable standard, and the absorbance of the blank medium served as the background. Data are expressed as the mean (± SD) of three data samples.

In the hypoxia treatment group, the cells were pre-treated with CoCl_2_ (100 μM) for 24 h to induce hypoxia, and then further incubated for 3 h in the presence or absence of MnO_2_ NPs. After washing three times, fresh cell culture media was added, and the cells were irradiated with X-rays (0, 1, 5, and 10 Gy) using the X-RAD 320 irradiator. Following incubation for 72 h, cell viability was measured as mentioned above.

#### Cell cycle analysis

To evaluate the effect of CoCl_2_ treatment on cell cycle, cells were seeded and cultured with or without CoCl_2_ (100 μM) for 24 h. Cells were harvested and re-suspended in 70% ethanol. The resuspended cells were fixed at 4°C for at least 2 h. RNase and propidium iodide (PI) were added for 30 min to stain the DNA. Cell cycle data were acquired and analyzed by flow cytometry (LSR Fortessa, BD Biosciences, San Jose, CA, USA).

#### Cell proliferation assay

The effect of CoCl_2_ treatment on the rate of cell proliferation was evaluated. Cells were seeded in 96-well plates at a density of 5,000 cells/well and incubated overnight for cell attachment. Cells were cultured with or without CoCl_2_ (100 μM) for 24 h. The culture media was replaced, and the cells were analyzed every 2 h for 96 h using an IncuCyte device (Essen BioScience, Ann Arbor, MI, USA). All experiments were carried out in triplicate.

#### Statistical analysis

The statistical significance of differences between groups was evaluated using Student's *t*-test. *P* < 0.05 indicated statistical significance.

## Results

**Figure 2:**
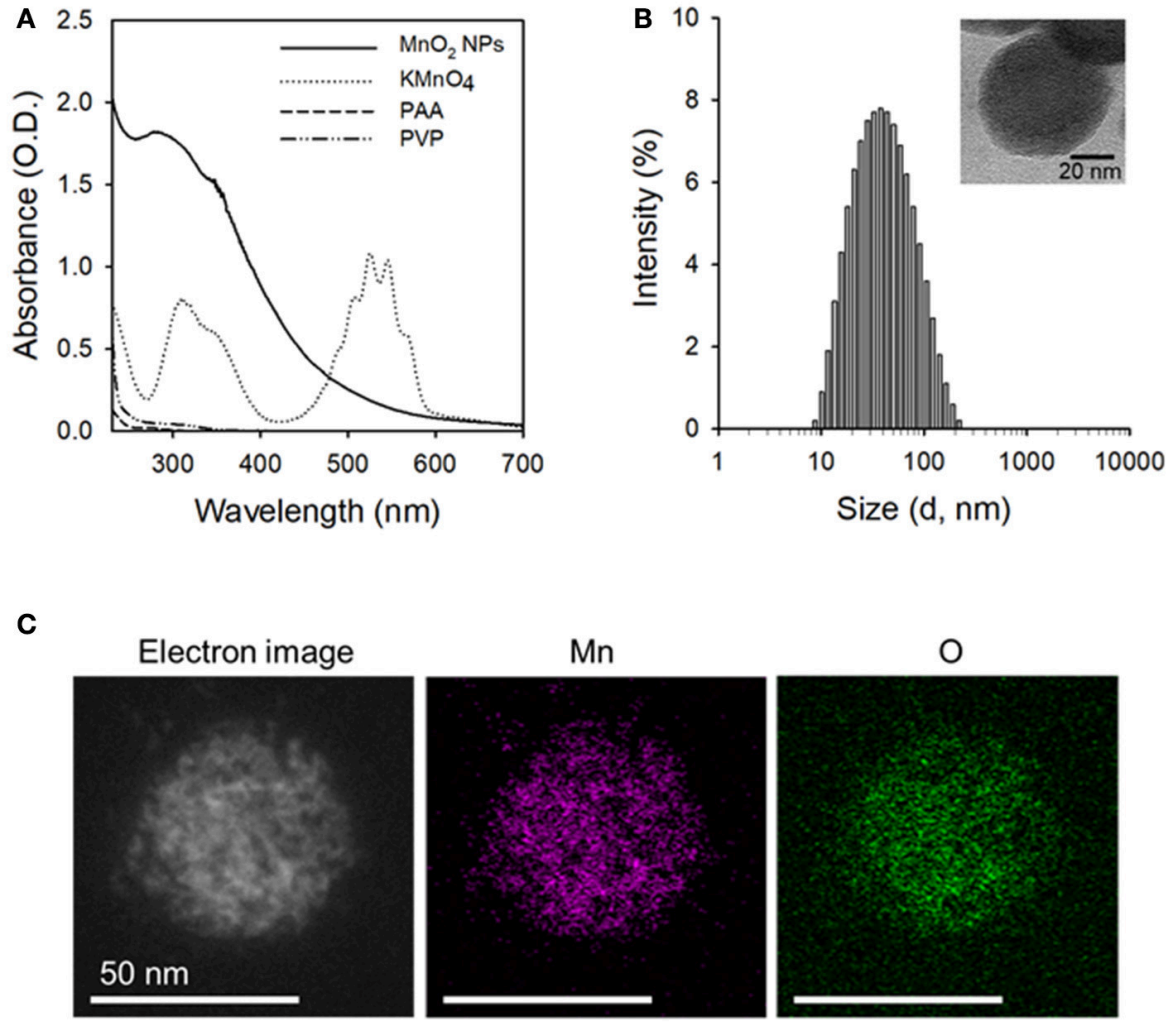
Characterization of the prepared MnO_2_ NPs. **(A)** UV/Vis absorption spectra of MnO_2_ NPs, KMnO_4_, PAA, and PVP. **(B)** Hydrodynamic size distribution of MnO_2_ NPs (inset: TEM image of MnO_2_ NPs). **(C)** Scanning TEM image and corresponding EDS element mapping analysis of Mn and O in MnO_2_ NPs.

MnO_2_ NPs were synthesized by reducing potassium permanganate (KMnO_4_) to manganese dioxide (MnO_2_) in the presence of biocompatible polymers (i.e., PVP and PAA). Based on our preliminary tests, the ratio between PVP and PAA was optimal at 1:1 wt/wt ratio because the highest yield of nanoparticles could be obtained at this ratio (data not shown). Formation of MnO_2_ NPs was confirmed based on the UV/Vis absorption spectra (Figure 2A) and TEM images (Figure 2B, inset and Figure S1A). As shown in Figure 2A, the KMnO_4_ peaks disappeared, whereas a new 300 nm peak appeared after formation of MnO_2_ NPs (Song et al., 2016). The hydrodynamic size and zeta potential of the prepared MnO_2_ NPs were 49.81 nm and −33.7 mV (polydispersity index: 0.275), respectively. Energy dispersive X-ray spectroscopy (EDS) mappings of Mn and O elements further demonstrate the homogeneous distribution of these elements in the MnO_2_ NPs (Figure 2C). Figure S1B shows the FT-IR spectra of MnO_2_ NPs, PVP, and PAA. The IR spectrum of MnO_2_ NPs showed characteristic Mn-O stretching vibration at 547.94 cm^−1^. Moreover, additional peaks at 3441.78, 2936.78, 1700.19, and 1289.18 cm^−1^ were observed, indicating the presence of PVP and PAA at the surface of MnO_2_ NPs. The peaks at 3441.78 cm^−1^ and 2936.78 cm^−1^ are due to contributions by O-H and C-H stretching vibrations, respectively. The peak at 1700.19 cm^−1^ can be assigned to the C = O stretching vibrations, and the peak at 1289.18 cm^−1^ is due to C-N stretching vibration from PVP. These results suggest that PVP and PAA were successfully attached to the surface of MnO_2_ NPs.

**Figure 3:**
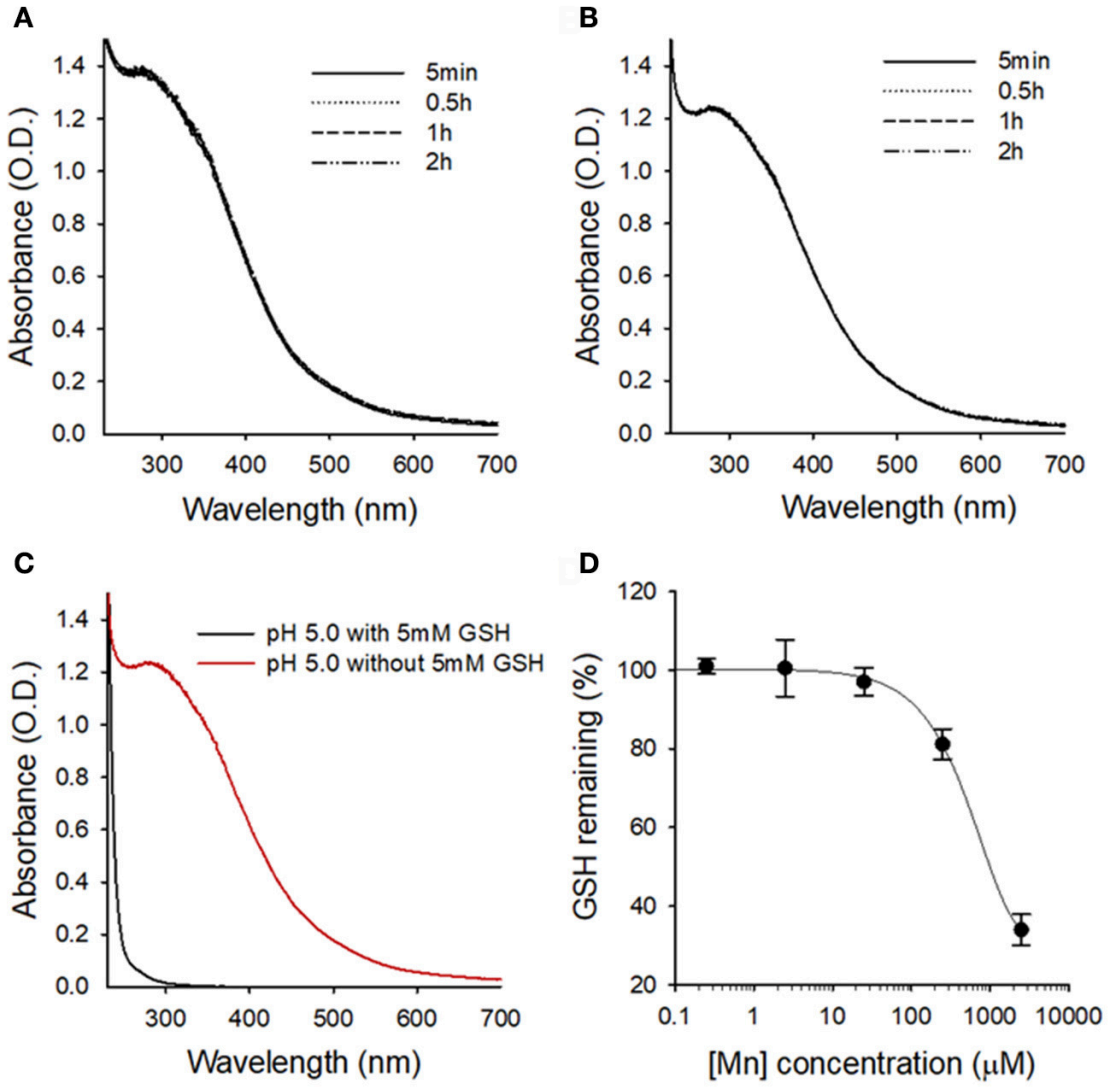
Stability of MnO_2_ NPs at different pH conditions and in the presence of GSH. UV/Vis absorption spectra of MnO_2_ NPs at indicated times at pH 7.4 **(A)** and pH 5.0 **(B)**. **(C)** Changes in UV/Vis absorption spectra of MnO_2_ NPs after 5 min of GSH treatment at pH 5.0. **(D)** Changes in the remaining GSH amount upon treatment with various concentrations of MnO_2_ NPs.

Next, we evaluated the stability of MnO_2_ NPs in buffer solutions of different pH values (pH 7.4 and 5.0), because previous studies have reported rapid dissolution of albumin-based MnO_2_ NPs at pH < 6.8 (Gordijo et al., 2015; Tian et al., 2017). We did not observe any changes in the UV/Vis absorption spectra of MnO_2_ NPs at pH 7.4 or 5.0 over 2 h (Figures 3A,B), indicating that the currently developed MnO_2_ NPs are stable at both physiological and lysosomal pH. Next, to confirm the reactivity of MnO_2_ NPs, we measured UV/Vis spectra of NPs in intracellular-mimicking environments. As the median concentration of GSH in intracellular space is known to be 5 mM (Estrela et al., 2006; Kim et al., 2016), the MnO_2_ NPs were incubated with 5 mM GSH at pH 5.0. Under these conditions, the characteristic absorption peak of MnO_2_ NPs rapidly disappeared (within 5 min, Figure 3C), indicating high reactivity of MnO_2_ NPs with GSH, and subsequent dissolution of the nanoparticles. Figure S2 shows the TEM images of GSH-treated MnO_2_ NPs. To further evaluate the GSH-reducing capability of MnO_2_ NPs, we incubated various concentrations of MnO_2_ NPs (0 μM, 0.25 μM, 2.5 μM, 25 μM, 250 μM, and 2.5 mM) with GSH (5 mM in pH 5.0), and measured the remaining GSH using a thiol detection assay (Figure 3D). We observed that the GSH concentration was reduced by approximately 70% by 2.5 mM of MnO_2_ NPs at pH 5.0.

**Figure 4:**
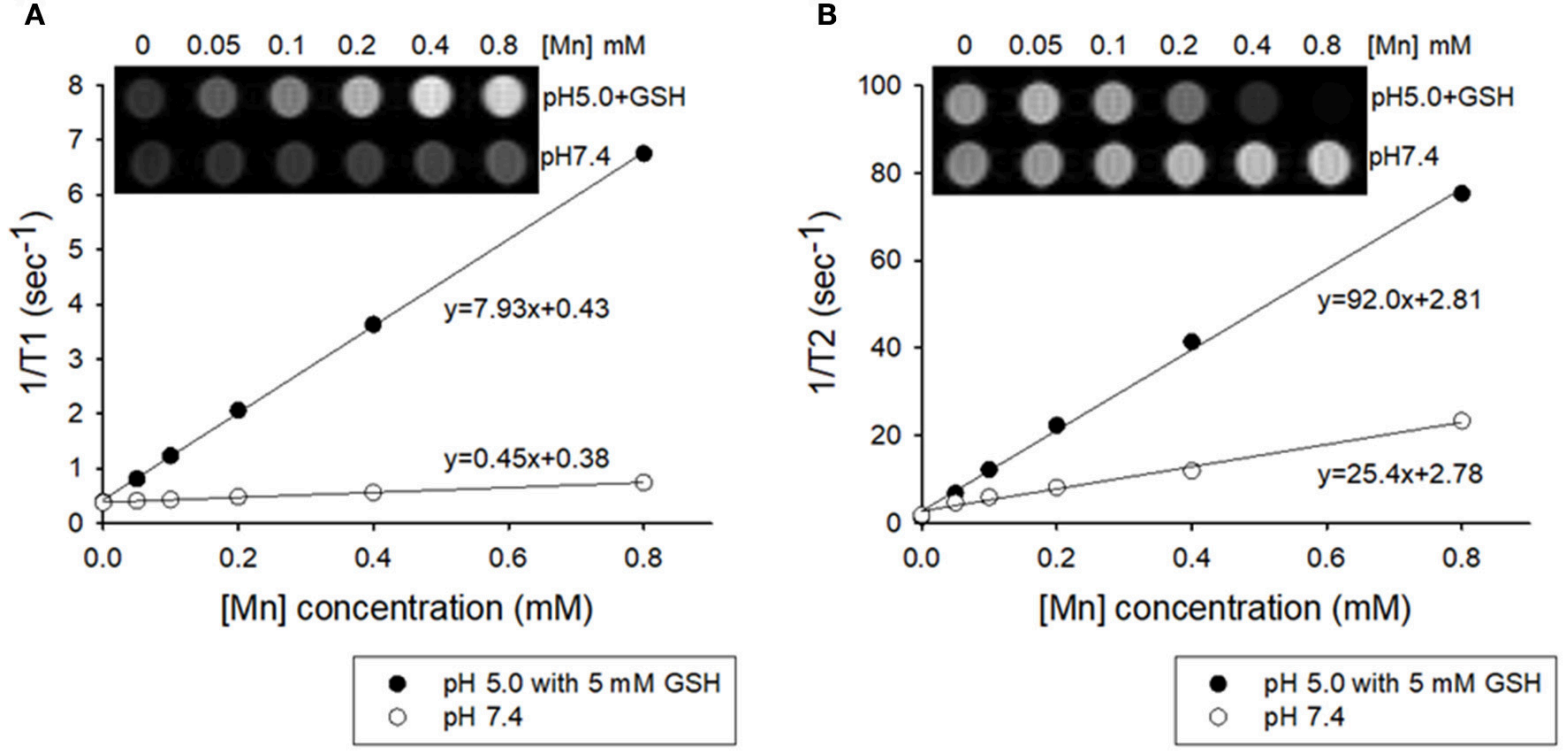
Plots of T1^−1^ and T2^−1^ vs. Mn concentration of MnO_2_ NPs in buffered solutions of pH 7.4 and pH 5.0 containing 5 mM GSH. **(A)** T1 relaxivity of the aqueous suspension of MnO_2_ NPs (inset: T1-weighted MRI). **(B)** T2 relaxivity of the aqueous suspension of MnO_2_ NPs (inset: T2-weighted MRI).

Manganese (II) compounds are promising candidates for clinical use as MRI contrast agents owing to their relatively high electronic spin and fast water exchange rate. In order to examine whether intracellular GSH could trigger enough dissolution of MnO_2_ NPs into Mn^2+^ for T1-weighted imaging, MRI of MnO_2_ NPs in acid or redox condition were further assessed. MnO_2_ NPs prepared in different solutions were measured on a 7.0-Tesla MR instrument. As shown in Figure 4A, the longitudinal relaxivity (*r*_1_) value of the MnO_2_ NPs at pH 5.0 containing 5 mM GSH was 7.93 mM^−1^s^−1^, which is 17.6-fold higher than the *r*_1_ value of MnO_2_ NPs at pH 7.4 (0.45 mM^−1^s^−1^). The T1-weighted images of MnO_2_ NPs rapidly whitened with increasing concentrations of NPs at pH 5.0 and 5 mM GSH. In addition, the transverse relaxivity (*r*_2_) value of MnO_2_ NPs also increased significantly in the presence of 5 mM GSH (Figure 4B, from 25.4 mM^−1^s^−1^ to 92.0 mM^−1^s^−1^). Meanwhile, MnCl_2_ aqueous solutions with different concentrations were selected as the positive control (*r*_1_ value = 7.38 mM^−1^s^−1^ and *r*_2_ value = 146.4 mM^−1^s^−1^ in Figure S3). These results suggest that the dissolution of MnO_2_ NPs inside cancer cells (i.e., under acidic and redox-inducing conditions) could function as the MRI contrast agent for both T1- and T2-weighted images.

**Figure 5:**
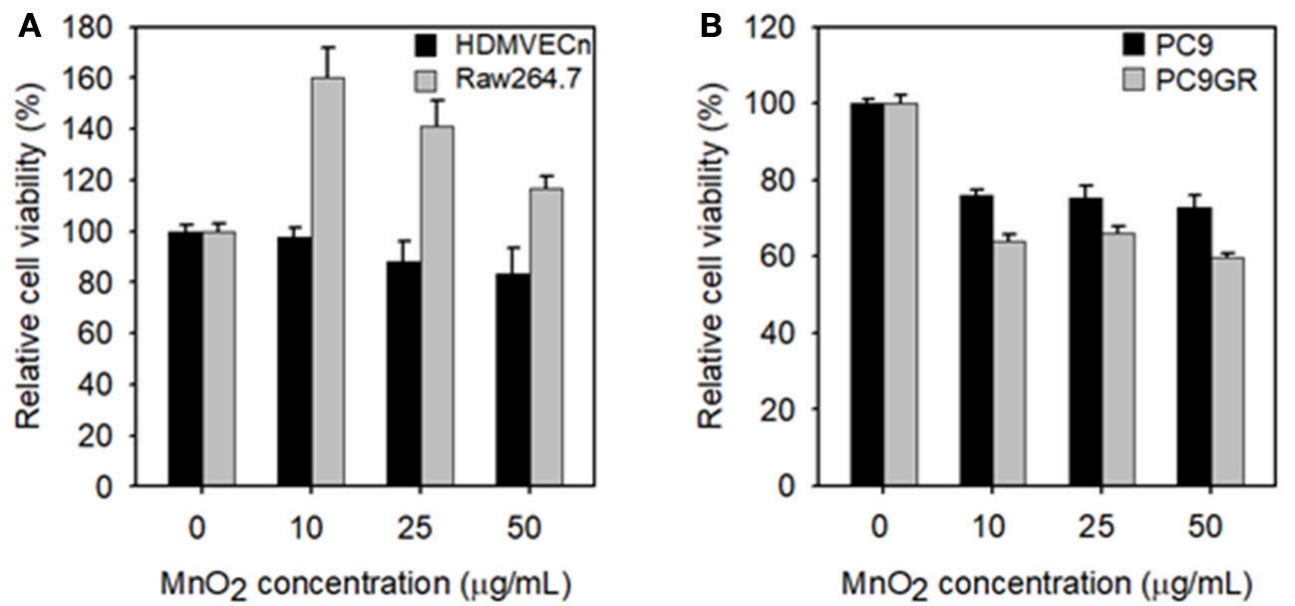
Measurements of cell viability of MnO_2_ NP-treated cells. **(A)** Cell viability of HDMVECn and Raw264.7 cells after MnO_2_ NP treatment. **(B)** Cell viability of PC9 and PC9GR cells after MnO_2_ NP treatment. Experiments were performed in triplicate for each condition.

Before we analyzed the therapeutic effect of MnO_2_ NPs, we tested the cytotoxic effect of MnO_2_ NPs on HDMVECn and Raw264.7 cells. The viability of HDMVECn cells was not significantly altered by any of the tested concentrations of MnO_2_ (Figure 5A). The Raw264.7 cells did not show any cytotoxic effects after MnO_2_ NP treatment; instead, they showed a substantial increase in cell numbers as the concentration increased from 0 to 50 μg/mL (*P* < 0.05) (Figure 5A). Next, we examined the effect of MnO_2_ NPs on NSCLC cells. Cell viability of PC9 and PC9GR lung cancer cells was measured after MnO_2_ NP treatment. Cell viability of both PC9 and PC9GR cell lines gradually decreased as MnO_2_ NP concentration increased. It is noteworthy that, when treated with MnO_2_ NPs at 50 μg/mL, the relative cell viabilities for PC9 and PC9GR cell lines dropped to 72.9 and 59.8% of the untreated control, respectively (*P* < 0.01) (Figure 5B). These results suggest that MnO_2_ NPs alone have a therapeutic effect on NSCLC cells.

**Figure 6:**
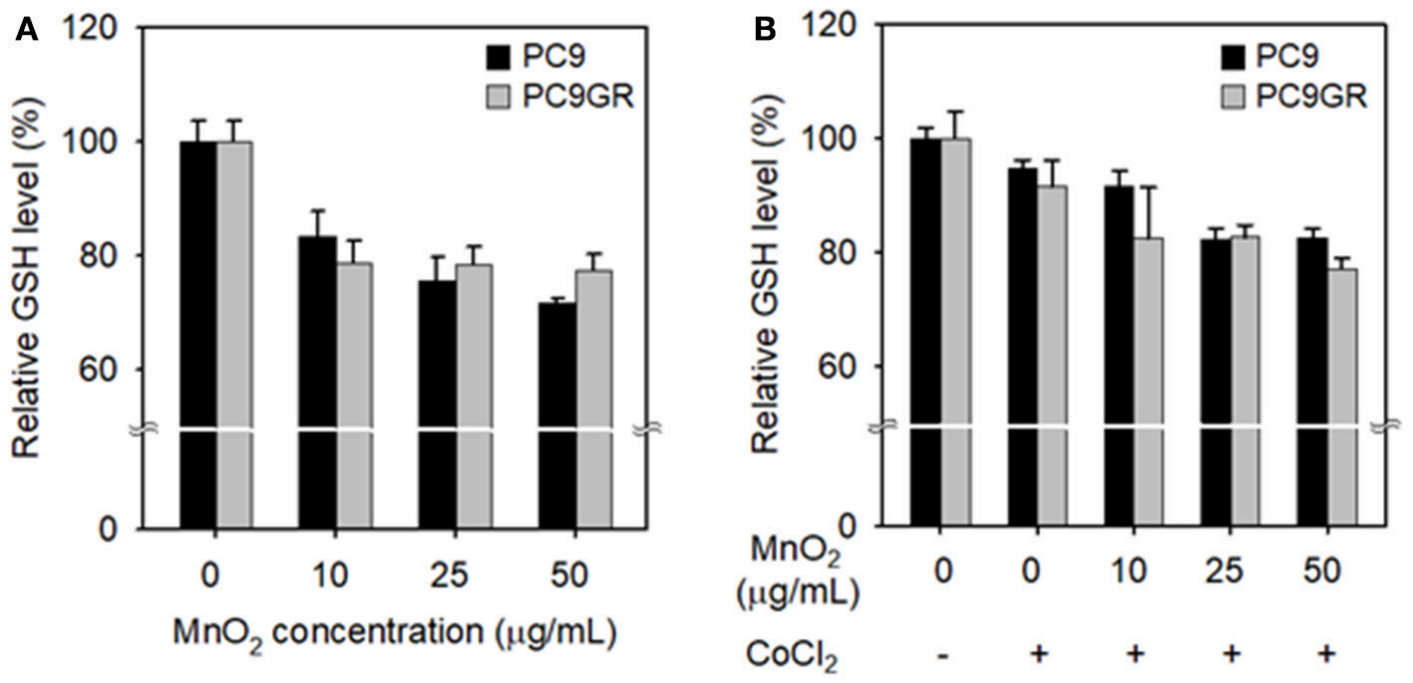
Changes in intracellular GSH levels after treatment with MnO_2_ NPs at various concentrations. GSH levels of cells incubated without **(A)** or with **(B)** CoCl_2_ for 24 h, and then treated with MnO_2_ NPs. Experiments were performed in triplicate for each condition.

We evaluated the effect of MnO_2_ NP treatment on the intracellular GSH levels in NSCLC cells. In both cell lines, relative intracellular GSH levels significantly decreased at MnO_2_ NP concentrations of 25–50 μg/mL when compared to the untreated control (*P* < 0.05). However, there was no further significant decrease when the MnO_2_ NP concentration was increased to 100 μg/mL (data not shown), and PC9GR had a higher GSH level than PC9 (Figure 6A). As many tumor masses have hypoxic conditions at their core, rather than normoxic conditions, we mimicked hypoxic conditions *in vitro* by incubating the cells with 100 μM of CoCl_2_. The cells were then treated with MnO_2_ NPs for 3 h, and intracellular GSH levels were measured. As shown in Figure 6B, the GSH levels in the MnO_2_ NP-treated cancer cells decreased in both cell lines; however, the pattern was slightly different from that of normoxic conditions (Figure 6A).

**Figure 7:**
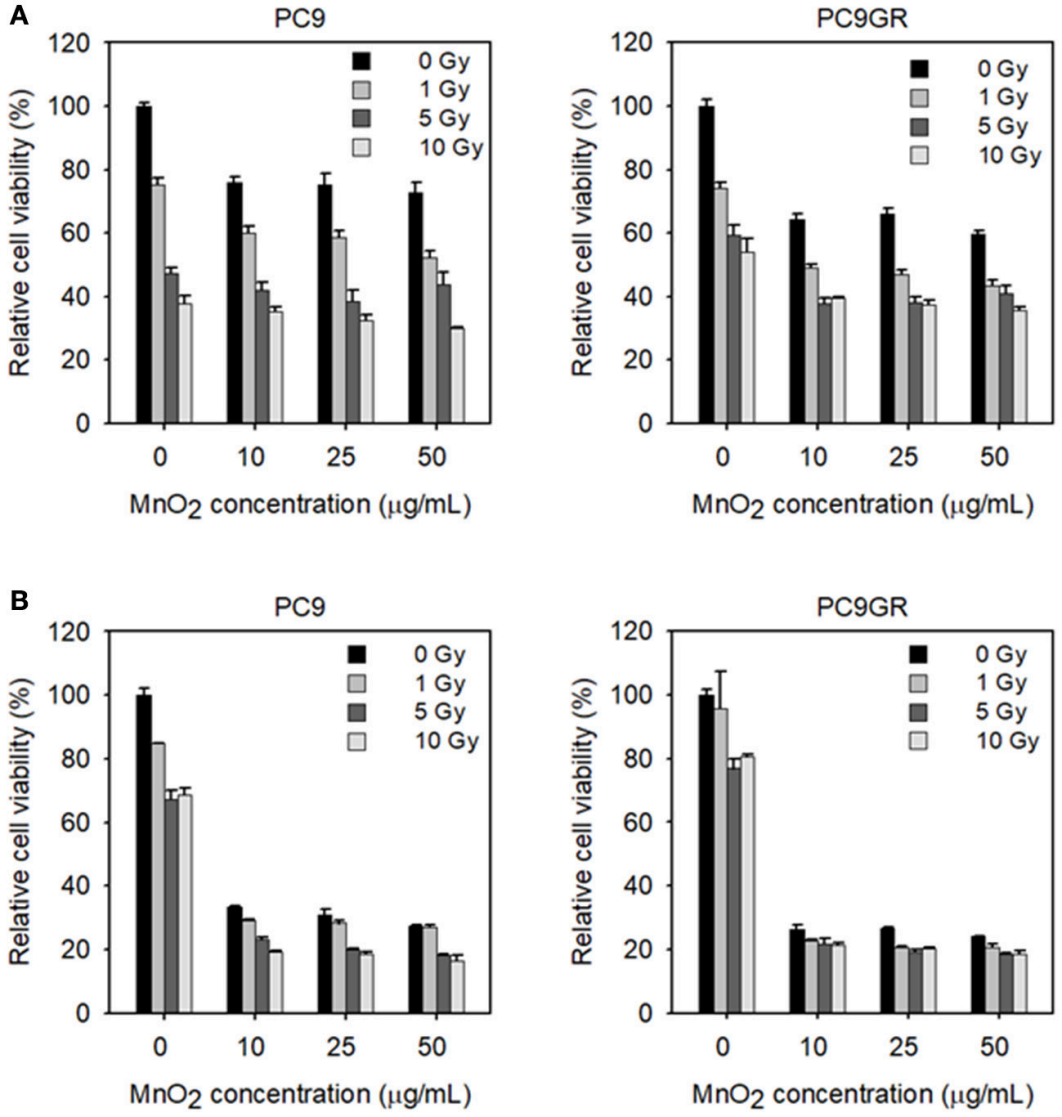
Viability of MnO_2_ NP-treated and X-ray-irradiated PC9 and PC9GR cells. **(A)** Cell viability of PC9 and PC9GR after MnO_2_ NP treatment and irradiation under normoxic condition. **(B)** Cell viability of PC9 and PC9GR after MnO_2_ NP treatment and irradiation after pretreatment with CoCl_2_. Experiments were performed in triplicate for each condition.

To examine the effect of MnO_2_ NPs on the viability of X-ray-irradiated cells, we measured cell viability with increasing MnO_2_ NP concentrations and X-ray irradiation. As expected, PC9GR showed more resistance to radiation therapy than its parent cell PC9 (*P* < 0.001) (Figure 7A). The viability of both PC9 and PC9GR cells decreased gradually with increase in both MnO_2_ NP concentration and X-ray irradiation dose, indicating that better therapeutic outcome could be achieved by combining MnO_2_ NP treatment and X-ray irradiation.

We then evaluated the effect of MnO_2_ NP treatment and X-ray irradiation under hypoxia-mimicking conditions. As shown in Figure 7B, both PC9 and PC9GR cells in hypoxic conditions became highly resistant to radiation therapy when compared to those in normoxic conditions (Figure 7A). In particular, PC9GR were highly resistant to radiation therapy, with the cell viability remaining around 80% of the untreated control even after X-ray irradiation at 10 Gy (Figure 7B). Interestingly, cells pretreated with CoCl_2_ and then treated with 10 μg/mL MnO_2_ NPs had markedly reduced viabilities of 33.3% for PC9 and 26.4% for PC9GR when compared to their normoxic condition counterparts (i.e., 76% for PC9 and 64% for PC9GR) (*P* < 0.001). Further reduction in cell viability could be obtained using X-ray irradiation.

**Figure 8:**
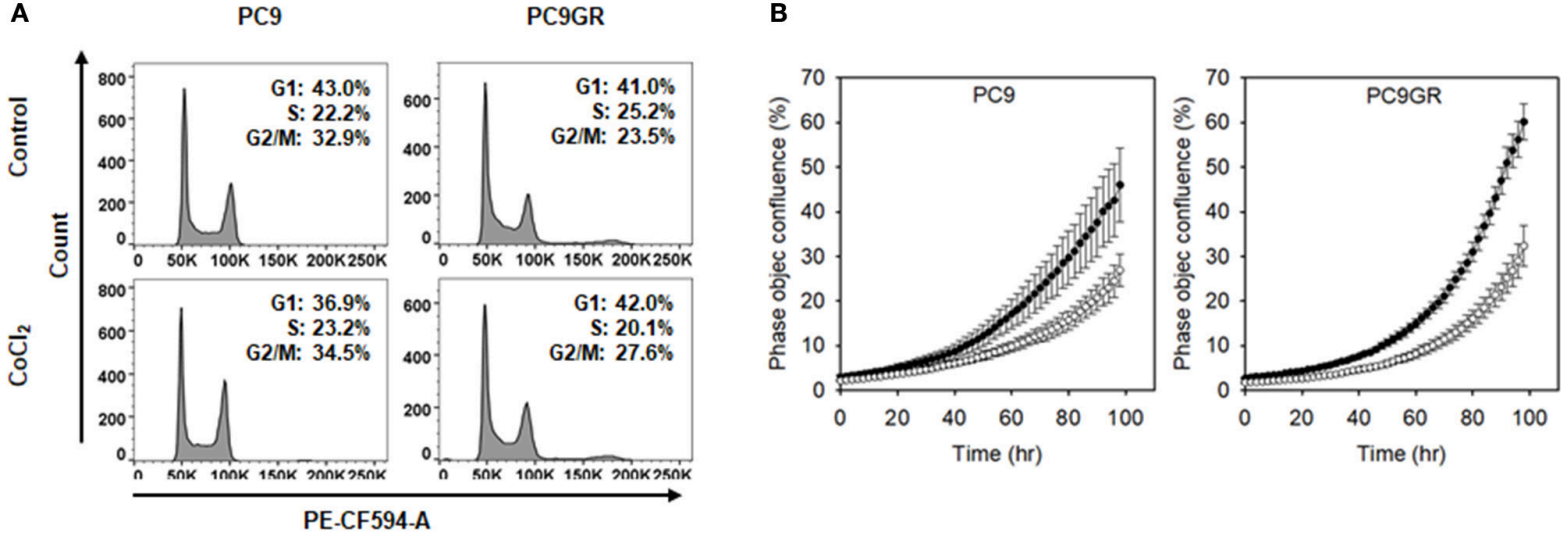
Cell cycle and proliferation of PC9 and PC9GR cells with or without CoCl_2_ treatment. **(A)** Flow cytometry of PC9 and PC9GR with CoCl_2_ treatment to analyzed cell cycle. **(B)** Measurement of occupied areas of PC9 and PC9GR cells with CoCl_2_ (°) or without CoCl_2_ (•). Error bars indicate standard deviation. Experiments were performed in triplicate for each condition.

To check whether the CoCl_2_ treatment resulted in cytotoxicity in the NSCLC cells, we analyzed the cell cycles of CoCl_2_ treated cells. Flow cytometry data showed no increase in the sub-G0/G1 population in either PC9 or PC9GR after CoCl_2_ treatment (Figure 8A), indicating that the decrease in cell viability in the CoCl_2_-preincubated and MnO_2_ NP-treated cells was not due to the cytotoxic effect of CoCl_2_. We also examined whether CoCl_2_ treatment affected the proliferation rate of PC9 or PC9GR cells. As shown in Figure 8B and Figure S4A, the rates of cell proliferation in both PC9 and PC9GR cells were halved upon CoCl_2_ treatment compared to those of the cells in normoxic conditions.

## Discussion

As mentioned above, NSCLC is a major cause of cancer-related deaths worldwide. Even though EGFR-TKIs therapy resulted in significant improvement in response rate and progression-free survival when compared to platinum-based conventional combination chemotherapy, most patients with EGFR-mutations treated with EGFR-TKIs acquired resistance within 9–14 months. Therefore, developing effective theranostic agents for the detection and therapy of NSCLC is still an ongoing challenge in clinics.

Recently, researchers have developed albumin-based MnO_2_ NPs for pH-responsive MR imaging and enhanced radiotherapy of breast cancers (Prasad et al., 2014; Gordijo et al., 2015; Abbasi et al., 2016; Song et al., 2016; Tian et al., 2017). MnO_2_ NPs were shown to have high reactivity toward endogenous hydrogen peroxide (H_2_O_2_) and protons, increasing the production of oxygen (O_2_) and acidity. These not only enabled pH-responsive MR imaging, but also modulated the tumor hypoxic status and subsequently enhanced radiotherapy *in vivo*.

Here, we synthesized new MnO_2_ NPs stabilized with PVP and PAA, and showed their potential utility for GSH-responsive MR imaging and enhanced therapy of NSCLC. MnO_2_ NPs developed in this study were shown to be highly stable at both physiological and acidic pH conditions. However, reaction with GSH resulted in rapid dissolution of the nanoparticles (Figure 3C) as well as reduction in GSH concentration in test solutions (Figure 3D), which resulted in a GSH-responsive turning on of MRI signals (Figure 4). Reduction in GSH levels after treatment of NSCLC cells with MnO_2_ NPs was also confirmed in the *in vitro* cell study (Figure 6). As the hydrodynamic size of MnO_2_ NPs was 49.81 nm, it is hypothesized that these nanoparticles could accumulate in tumor tissues through the enhanced permeability and retention (EPR) effect (Hashizume et al., 2000; Fang et al., 2011), be internalized *via* endocytosis, and then, once inside the cancer cells, reduced to Mn^2+^ ions by intracellular GSH, thereby enabling selective MR imaging of tumors.

We demonstrated that MnO_2_ NPs did not affect viability of normal cells at the tested concentrations (i.e., 0–50 μg/mL), supporting the biocompatibility of the prepared nanoparticles. Interestingly, treatment of NSCLC cells (i.e., PC9 and PC9GR) with MnO_2_ NPs caused significant cytotoxic effects, which we did not expect. Moreover, even though these NSCLC cells became highly resistant to radiation therapy under hypoxic conditions, their viabilities drastically dropped to 33.3% (PC9) and 26.4% (PC9GR) when treated with 10 μg/mL MnO_2_ NPs. Additional dose-dependent therapeutic effects could be obtained upon X-ray irradiation. To check whether the hypoxic conditions led to cell death, we treated cells with CoCl_2_ and analyzed proliferation and cell cycle. We found no change in cell cycle populations and only observed retarded cell proliferation rate in hypoxic conditions. These results coincided with a previous report stating that cell proliferation-cyclin proteins are reduced under hypoxic conditions (Cogo et al., 2003). We also checked the level of cyclin D1 protein and found that it was reduced by CoCl_2_ treatment (Figure S4B). Thus, CoCl_2_ treatment itself did not induce cell death, but it might have a marked effect on cell viability in the presence of MnO_2_ NPs. Therefore, MnO_2_ NPs might have immense potential for treating EGFR-TKI-resistant NSCLC in both normoxic and hypoxic conditions. Currently, we are investigating the mechanisms of action to determine how NSCLC cells could be effectively treated by MnO_2_ NPs.

## Conclusions

In this study, we showed that one of the major challenges of EGFR TKI-resistant lung cancer treatment can be overcome by MnO_2_ NPs, which are effective at reducing GSH levels. The application of radiation therapy in combination with MnO_2_ NPs can effectively induce cancer cell death compared to radiation alone. In addition, the released Mn^2+^ ions from MnO_2_ NPs in tumor environments provide potential for them to be used as GSH-responsive T1- and T2-weighted MRI contrast agents.

## Author contributions

MHC: Performed the synthesis and characterization of MnO_2_ nanoparticles and wrote the manuscript; E-SC: Performed *in vitro* cell experiments and wrote the manuscript; SK: Synthesized the MnO_2_ nanoparticles; S-HG: Supervised the *in vitro* experiments and wrote the manuscript; YC: Designed and supervised all the experiments and revised the manuscript.

### Conflict of interest statement

The authors declare that the research was conducted in the absence of any commercial or financial relationships that could be construed as a potential conflict of interest.

## References

[B1] Abbasi A. Z. Gordijo C. R. Amini M. A. Maeda A. Rauth A. M. DaCosta R. S.. (2016). Hybrid manganese dioxide nanoparticles potentiate radiation therapy by modulating tumor hypoxia. Cancer Res. 76, 6643–6656. 10.1158/0008-5472.CAN-15-3475 27758881

[B2] Bump E. A. Brown J. M. (1990). Role of glutathione in the radiation response of mammalian cells *in vitro* and *in vivo*. Pharmacol. Ther. 47, 117–136. 10.1016/0163-7258(90)90048-7 2195553

[B3] Chen Y. Chen H. Zhang S. Chen F. Sun S. He Q.. (2012a). Structure-property relationships in manganese oxide - mesoporous silica nanoparticles used for T1-weighted MRI and simultaneous anti-cancer drug delivery. Biomaterials 33, 2388–2398. 10.1016/j.biomaterials.2011.11.086 22177841

[B4] Chen Y. Yin Q. Ji X. Zhang S. Chen H. Zheng Y.. (2012b). Manganese oxide-based multifunctionalized mesoporous silica nanoparticles for pH-responsive MRI, ultrasonography and circumvention of MDR in cancer cells. Biomaterials 33, 7126–7137. 10.1016/j.biomaterials.2012.06.059 22789722

[B5] Cogo A. Napolitano G. Michoud M. C. Barbon D. R. Ward M. Martin J. G. (2003). Effects of hypoxia on rat airway smooth muscle cell proliferation. J. Appl. Physiol. 94, 1403–1409. 10.1152/japplphysiol.00363.2002 12626471

[B6] Deng R. Xie X. Vendrell M. Chang Y. T. Liu X. (2011). Intracellular glutathione detection using MnO_2_-nanosheet-modified upconversion nanoparticles. J. Am. Chem. Soc. 133, 20168–20171. 10.1021/ja2100774 22107163

[B7] Estrela J. M. Ortega A. Obrador E. (2006). Glutathione in cancer biology and therapy. Crit. Rev. Clin. Lab. Sci. 43, 143–181. 10.1080/10408360500523878 16517421

[B8] Fang J. Nakamura H. Maeda H. (2011). The EPR effect: unique features of tumor blood vessels for drug delivery, factors involved, and limitations and augmentation of the effect. Adv. Drug Deliv. Rev. 63, 136–151. 10.1016/j.addr.2010.04.009 20441782

[B9] Fitzmaurice C. Allen C. Barber R. M. Barregard L. Bhutta Z. A. Brenner H.. (2017). Global, regional, and national cancer incidence, mortality, years of life lost, years lived with disability, and disability-adjusted life-years for 32 cancer groups, 1990 to 2015. JAMA Oncol. 3, 524–548. 10.1001/jamaoncol.2016.5688 27918777 PMC6103527

[B10] Gordijo C. R. Abbasi A. Z. Amini M. A. Lip H. Y. Maeda A. Cai P. (2015). Design of hybrid MnO_2_-polymer-lipid nanoparticles with tunable oxygen generation rates and tumor accumulation for cancer treatment. Adv. Funct. Mater. 25, 1858–1872. 10.1002/adfm.201404511

[B11] Hao Y. Wang L. Zhang B. Li D. Meng D. Shi J.. (2016). Manganese dioxide nanosheets-based redox/pH-responsive drug delivery system for cancer theranostic application. Int. J. Nanomed. 11, 1759–1778. 10.2147/IJN.S98832 27199556 PMC4857809

[B12] Hashizume H. Baluk P. Morikawa S. McLean J. W. Thurston G. Roberge S.. (2000). Openings between defective endothelial cells explain tumor vessel leakiness. Am. J. Pathol. 156, 1363–1380. 10.1016/S0002-9440(10)65006-7 10751361 PMC1876882

[B13] Herbst R. S. Shin D. M. (2002). Monoclonal antibodies to target epidermal growth factor receptor-positive tumors: a new paradigm for cancer therapy. Cancer 94, 1593–1611. 10.1002/cncr.10372 11920518

[B14] Herszage J. Afonso M. S. (2003). Oxidation of cysteine and glutathione by soluble polymeric MnO_2_. Environ. Sci. Technol. 37, 3332–3338. 10.1021/es0340634 12966978

[B15] Kim J. Chae J. Kim J. S. Goh S. H. Choi Y. (2016). Photosensitizer-conjugated tryptophan-containing peptide ligands as new dual-targeted theranostics for cancers. Int. J. Pharm. 513, 584–590. 10.1016/j.ijpharm.2016.09.071 27686051

[B16] Koczkur K. M. Mourdikoudis S. Polavarapu L. Skrabalak S. E. (2015). Polyvinylpyrrolidone (PVP) in nanoparticle synthesis. Dalton Trans. 44, 17883–17905. 10.1039/C5DT02964C 26434727

[B17] Lee J. M. Lee W. H. Kay H. Y. Kim E. S. Moon A. Kim S. G. (2012). Hemin, an iron-binding porphyrin, inhibits HIF-1α induction through its binding with heat shock protein 90. Int. J. Cancer 130, 716–727. 10.1002/ijc.26075 21413014

[B18] Maemondo M. Inoue A. Kobayashi K. Sugawara S. Oizumi S. Isobe H. (2010). Gefitinib or chemotherapy for non-small-cell lung cancer with mutated EGFR. N. Engl. J. Med. 362, 2380–2388. 10.1056/NEJMoa0909530 20573926

[B19] Mitsudomi T. Morita S. Yatabe Y. Negoro S. Okamoto I. Tsurutani J. (2010). Gefitinib versus cisplatin plus docetaxel in patients with non-small-cell lung cancer harbouring mutations of the epidermal growth factor receptor (WJTOG3405): an open label, randomized phase 3 trial. Lancet Oncol. 11, 121–128. 10.1016/S1470-2045(09)70364-X 20022809

[B20] Mok T. S. Wu Y. L. Thongprasert S. Yang C. H. Chu D. T. Saijo N. (2009). Gefitinib or carboplatinpaclitaxel in pulmonary adenocarcinoma. N. Engl. J. Med. 361, 947–957. 10.1056/NEJMoa0810699 19692680

[B21] Morgillo F. Della Corte C. M. Fasano M. Ciardiello F. (2016). Mechanisms of resistance to EGFR-targeted drugs: lung cancer. ESMO Open 1:e000060. 10.1136/esmoopen-2016-000060 27843613 PMC5070275

[B22] Prasad P. Gordijo C. R. Abbasi A. Z. Maeda A. Ip A. Rauth A. M. (2014). Multifunctional albumin-MnO_2_ nanoparticles modulate solid tumor microenvironment by attenuating hypoxia, acidosis, vascular endothelial growth factor and enhance radiation response. ACS Nano 8, 3202–3212. 10.1021/nn405773r 24702320

[B23] Rapisarda A. Melillo G. (2010). Combination strategies targeting hypoxia inducible factor (HIF-1) for cancer therapy, in The Tumor Microenvironment, ed Bagley R. (New York, NY: Springer), 3–21.

[B24] Song M. Liu T. Shi C. Zhang X. Chen X. (2016). Bioconjugated manganese dioxide nanoparticles enhance chemotherapy response by priming Tumor-Associated Macrophages toward M1-like phenotype and attenuating tumor hypoxia. ACS Nano 10, 633–647. 10.1021/acsnano.5b06779 26650065 PMC5242343

[B25] Teicher B. A. (1995). Physiologic mechanisms of therapeutic resistance. Blood flow and hypoxia. Hematol. Oncol. Clin. North Am. 9, 475–506. 7642474

[B26] Tian L. Chen Q. Yi X. Chen J. Liang C. Chao Y.. (2017). Albumin-templated manganese dioxide nanoparticles for enhanced radioisotope therapy. Small 13:1700640. 10.1002/smll.201700640 28544324

[B27] Vaupel P. Mayer A. (2007). Hypoxia in cancer: significance and impact on clinical outcome. Cancer Metastasis Rev. 26, 225–239. 10.1007/s10555-007-9055-1 17440684

[B28] Wangari-Talbot J. Hopper-Borge E. (2013). Drug resistance mechanisms in non-small cell lung carcinoma. J. Can. Res. Updates 2, 265–282. 24634705 10.6000/1929-2279.2013.02.04.5 PMC3952141

[B29] Wu D. Yotnda P. (2011). Induction and testing of hypoxia in cell culture. J. Vis. Exp. 12:e2899 10.3791/2899 PMC3217626 21860378

[B30] Yan X. Song Y. Zhu C. Song J. Du D. Su X.. (2016). Graphene quantum dot-MnO_2_ nanosheet based optical sensing platform: a sensitive fluorescence “turn off-on” nanosensor for glutathione detection and intracellular imaging. ACS Appl. Mater. Interfaces 8, 21990–21996. 10.1021/acsami.6b05465 27494553

