# Supplementary material for: Redox-Responsive Manganese Dioxide Nanoparticles for Enhanced MR Imaging and Radiotherapy of Lung Cancer
Source: Front Chem. 2017 Dec 4;5:109. doi: 10.3389/fchem.2017.00109 (PMC5722798; doi:10.3389/fchem.2017.00109)
Supplement: Supplementary file 1 [file DataSheet1.docx]

Supplementary Material

**Redox-responsive manganese dioxide nanoparticles for enhanced MR imaging and radiotherapy of lung cancer**

Mi Hyeon Cho, Eun-Seok Choi, Sehee Kim, Sung-Ho Goh* and Yongdoo Choi*

*** Correspondence:** Yongdoo Choi: ydchoi@ncc.re.kr; Sung-Ho Goh: andrea@ncc.re.kr

##
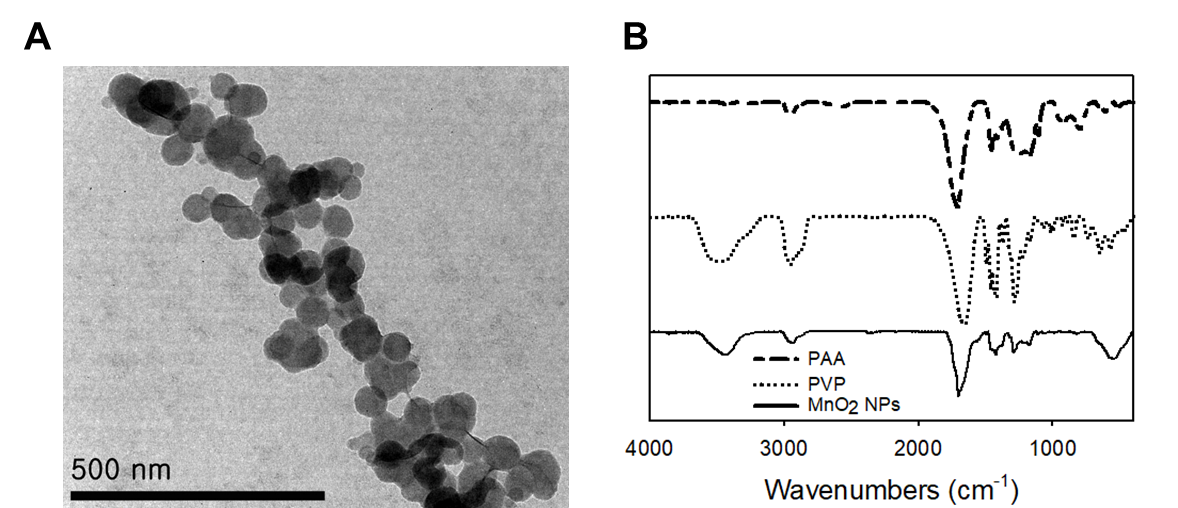


**Supplementary Figure 1.** (A) TEM image of MnO_2_ nanoparticles. (B) FT-IR spectra of PAA, PVP, and MnO_2_ NPs.


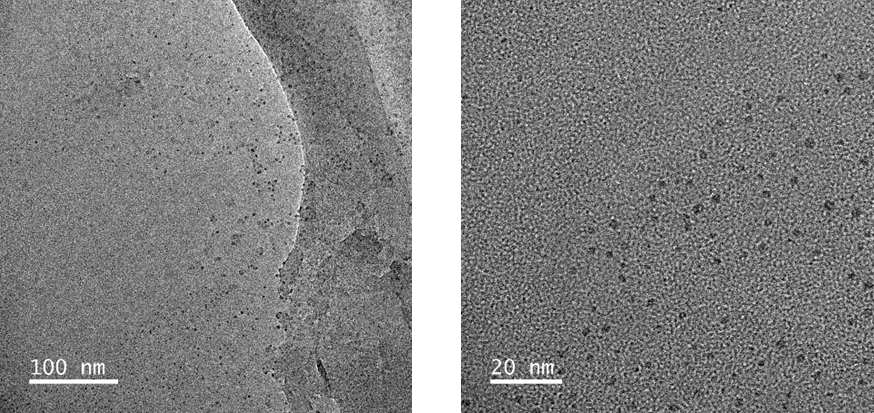


**Supplementary Figure 2.** TEM images of MnO_2_ NPs dispersed in acetate buffer (pH 5.0, 0.1 M) containing 5 mM GSH.


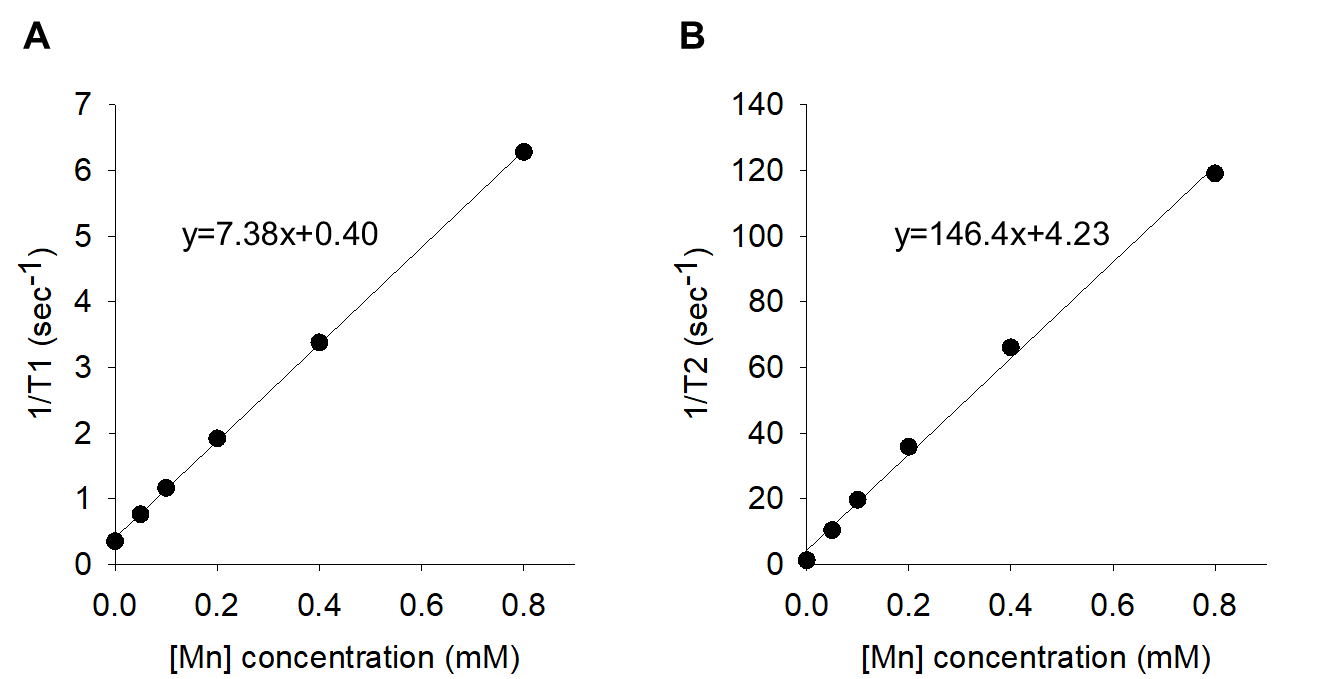


**Supplementary Figure 3.** Plots of (A) T1^-1^ and (B) T2^-1^ versus Mn concentration of MnCl_2_. MnCl_2_ was used as a positive control.


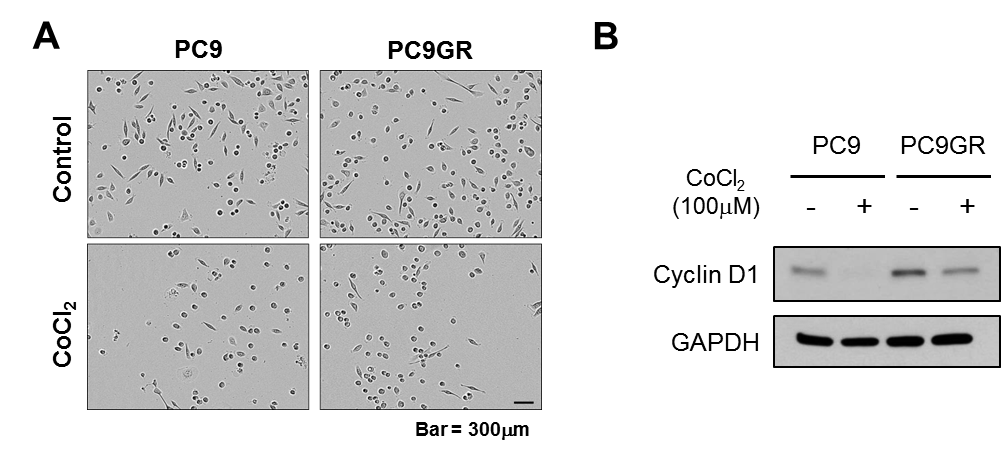


**Supplementary Figure 4.** (A) Proliferation status of PC9 and PC9GR with or without CoCl_2_ treatment. Representative cell images were acquired by IncuCyte at 72 h. bar = 300 μm. (B) Cyclin D1 protein levels in PC9 and PC9GR with or without CoCl_2_ treatment. GAPDH was used as the loading control.
